# Shoulder Injury Related to Vaccine Administration (SIRVA) Following COVID-19 Vaccination: Correlating MRI Findings with Patient Demographics

**DOI:** 10.3390/tomography11050053

**Published:** 2025-05-02

**Authors:** Naser Obeidat, Ruba Khasawneh, Ahmad Alrawashdeh, Ali M. Abdel Kareem, Mohammad K. Al-na’asan, Mohammad Alkhatatba, Suhaib Bani Essa

**Affiliations:** 1 Department of Diagnostic Radiology and Nuclear Medicine, Faculty of Medicine, Jordan University of Science and Technology, Irbid 22110, Jordan; rakhasawneh2@just.edu.jo (R.K.); amabdelkareem20@med.just.edu.jo (A.M.A.K.); mkalnaasan23@med.just.edu.jo (M.K.A.-n.); 2 Department of Allied Medical Sciences, Faculty of Applied Medical Sciences, Jordan University of Science and Technology, Irbid 22110, Jordan; aaalrawashdeh@just.edu.jo; 3 Department of Special Surgery, Faculty of Medicine, Jordan University of Science and Technology, Irbid 22110, Jordan; maalkhatatba@just.edu.jo (M.A.); smbaniessa@just.edu.jo (S.B.E.)

**Keywords:** COVID-19 vaccine, MRI, rotator cuff, shoulder, SIRVA, subacromial bursitis, vaccine

## Abstract

Objectives: Shoulder injury related to vaccine administration (SIRVA), previously observed with influenza vaccines, has gained clinical significance with widespread COVID-19 vaccination. However, few studies correlate vaccine types and demographic factors with the MRI findings of SIRVA. This study aimed to evaluate MRI findings of SIRVA following COVID-19 vaccination and assess associations with vaccine type and patient characteristics. Methods: A retrospective cohort study was conducted on 35 patients with new-onset shoulder complaints within six weeks of COVID-19 vaccination between May 2021 and May 2022. MRI findings suggestive of SIRVA were reviewed, including subacromial bursitis, rotator cuff tears, and adhesive capsulitis. Demographic data, vaccine type, clinical symptoms, and treatments were collected. Follow-up interviews (1–30 September 2024) assessed symptom persistence and vaccine hesitancy. Descriptive statistics and Chi-square tests were used to explore associations. Results: Of the 35 patients (mean age 53.6 ± 9.0 years; 54.3% female), subacromial bursitis was the most common MRI finding (89.5%), followed by tendonitis (47.4%) and adhesive capsulitis (36.8%). Tendonitis correlated with older age (*p* = 0.024) and AstraZeneca vaccination (*p* = 0.033). Subacromial bursitis was linked to female sex (*p* = 0.013) and higher BMI (*p* = 0.023). Adhesive capsulitis was associated with receiving the Sinopharm vaccine (*p* = 0.029). Persistent symptoms (22.9%) were more common in younger patients, women, and those with right-sided injections. Conclusions: SIRVA following COVID-19 vaccination showed different MRI patterns associated with female sex, higher BMI, and vaccine type. Awareness of these patterns may expedite recognition of COVID-19-associated SIRVA in routine practice.

## 1. Introduction

Shoulder injury related to vaccine administration (SIRVA) is a rare yet clinically significant complication associated with improper injection technique. Instead of being deposited into the mid-deltoid muscle or subcutaneous tissue, the vaccine may be inadvertently delivered into periarticular structures, principally the subacromial-subdeltoid bursa, thereby precipitating an inflammatory cascade with subsequent damage to tendons, ligaments, and bursae [1]. Although first widely recognized in the context of influenza vaccination, SIRVA has garnered increased attention following the global mass administration of COVID-19 vaccines [2]. Mackenzie and colleagues reported an estimated incidence of two cases per ten-million COVID-19 vaccine doses administered, underscoring the rarity but also the potential underdiagnosis of this entity [3].

Certain demographic and anatomical characteristics appear to elevate the risk of SIRVA. Females exhibit a higher incidence, possibly due to smaller deltoid mass or lower muscle density, which can make proper intramuscular needle placement more challenging [4]. Furthermore, individuals with pre-existing shoulder pathologies, such as rotator cuff tears, bursitis, or tendinopathy, may be more vulnerable to inadvertent needle trauma [5]. Clinically, patients with SIRVA typically present with localized shoulder pain and restricted motion within 48 h of vaccination. These symptoms often overlap with other distinct shoulder pathologies (i.e., most commonly, subacromial bursitis, adhesive capsulitis AC (frozen shoulder), or rotator cuff tears), leading to diagnostic complexity [6,7]. A structured evaluation is therefore critical to differentiate SIRVA from more routine causes of shoulder pain and ensure timely intervention.

Imaging plays a pivotal role in the prompt and accurate diagnosis of SIRVA. While ultrasound can help detect superficial soft tissue or bursal abnormalities, magnetic resonance imaging (MRI) provides superior visualization of deeper structures, including tendons, joint capsules, and bone marrow changes [6]. Hence, MRI not only facilitates an earlier and more precise diagnosis but also guides management, particularly in delineating the extent of rotator cuff disease or adhesive capsulitis. Early detection and appropriate treatment, ranging from conservative measures, such as physical therapy and nonsteroidal anti-inflammatory drugs, to possible surgical interventions, can mitigate long-term morbidity and optimize functional recovery.

Given the expanding use of various COVID-19 vaccine formulations worldwide and the inherent risk of inadvertent injection into shoulder structures, there is a pressing need to identify and characterize MRI findings specifically associated with SIRVA. Moreover, data correlating imaging features with vaccine types and demographic variables remain limited. Therefore, the present study aims to investigate the MRI features of SIRVA following COVID-19 vaccination in a sizable patient cohort, correlating imaging findings with age, sex, comorbidities, and potential associations with different vaccine types, while recognizing this study’s exploratory nature.

## 2. Materials and Methods

### 2.1. Study Design

This study employed a retrospective cohort approach to identify and examine individuals who received COVID-19 vaccines and subsequently developed shoulder complaints consistent with SIRVA. This study was approved by the Institutional Review Board of the King Abdullah University Hospital/Jordan University of Science and Technology (Approval No. 9/177/2024). In accordance with the Declaration of Helsinki, this retrospective observational investigation did not require written informed consent because it relied on existing clinical and imaging records. Confidentiality was maintained by assigning unique identifiers to each participant and all data were securely stored with access restricted to authorized research personnel.

### 2.2. Participants

All adult patients (≥18 years of age) presenting to the shoulder orthopedic clinic between 1 May 2021 and 1 May 2022 were screened. Inclusion criteria required new-onset shoulder pain or dysfunction within six weeks post-vaccination and availability of a diagnostic MRI study for the symptomatic shoulder. Exclusion criteria comprised any documented pre-existing shoulder pathology (e.g., rotator cuff tears, advanced osteoarthritis) or recent trauma prior to vaccination and MRI images of insufficient diagnostic quality. Two researchers independently reviewed the medical records; any disagreement regarding eligibility was resolved through consensus to minimize selection bias.

### 2.3. Data Collection

Demographic and clinical data were extracted from patient records, including age, gender, weight, height, occupation, physical activity, diabetes status, vaccine type, number of doses, side of injection, time interval to MRI, symptom onset/duration, and administered treatments. Baseline shoulder MRI was unavailable because pre-vaccination imaging is not standard practice; instead, this retrospective design captured all consecutive cases presenting during the vaccination campaign. Missing or ambiguous values (e.g., symptom onset date) were coded as “unknown” and retained for transparent reporting.

### 2.4. MRI Protocol

All MRIs were performed on 1.5 T or 3 T Philips machines (Philips Ingenia or Achieva, respectively; Best, The Netherlands) using a dedicated shoulder coil. The imaging protocol included T1-weighted, T2-weighted, and proton density fat-saturated sequences in axial, coronal oblique, and sagittal oblique planes with a 3 mm slice thickness, a 0.3 mm interslice gap, and a 320 × 223 imaging matrix. No intravenous contrast was administered in accordance with our institution’s standard practice for the shoulder joint.

Examinations featuring severe artifacts that hindered interpretation were excluded. Two musculoskeletal radiologists (each with ≥5 years of experience) independently evaluated the images for entheseal erosions, bone marrow and muscle edema, avascular necrosis, cartilage defects, tendinopathy, AC (edema/thickening of the rotator cuff interval and/or axillary capsule), subacromial bursitis, effusion, and rotator cuff tears. Discrepancies were resolved through a joint re-evaluation. While both radiologists were aware that these patients had post-vaccination shoulder complaints, specific vaccine type details were not systematically revealed during interpretation. However, no formal inter-rater reliability statistics (e.g., kappa) were calculated, which could have quantified the reproducibility of our findings.

### 2.5. Follow-Up and Outcome Measures

Between 1 and 30 September 2024, phone interviews were conducted using a standardized questionnaire to ascertain whether patients continued to experience symptoms. Persistent symptoms were defined as those lasting more than three months post-vaccination, including pain, restricted range of motion, or functional impairment. A subset of patients underwent repeat MRI for clinical reasons and initial and follow-up imaging were compared to detect changes.

### 2.6. Statistical Analysis

Data were imported into Microsoft Excel, edited, and then analyzed using Stata Statistical Software version 16.0 (StataCorp LLC., College Station, TX, USA). Descriptive analyses were conducted for continuous variables (means, standard deviations) and categorical data (frequencies, percentages). The normality of continuous variables was confirmed, t-tests were applied to compare means, and Chi-square or Fisher’s exact tests were used for categorical comparisons, depending on cell counts. Statistical significance was set at *p* < 0.05. Because this is an exploratory study, we did not apply formal corrections for multiple comparisons (e.g., Bonferroni, FDR). Consequently, these *p*-values should be interpreted with caution as some associations may lose significance upon more stringent adjustment.

## 3. Results

### 3.1. Patient Demographics

The study included 35 patients diagnosed with SIRVA following COVID-19 vaccination. Table 1 presents the demographics and vaccination characteristics of the study patients. The mean age was 53.6 ± 9.0 years and 54.3% were younger than 55 years. There was a slight female predominance (54.3%, *n* = 19). The mean body mass index (BMI) was 28.4 ± 4.8 kg/m^2^, with 80% (*n* = 28) of patients classified as overweight or obese. Diabetes mellitus was present in 34.3% of the patients. The majority of patients (54.3%, *n* = 29) did not engage in regular physical activity while 28.6% (*n* = 10) exercised occasionally and 17.1% (*n* = 6) reported regular physical activity.

The Pfizer vaccine was the most administered, received by 57.1% (*n* = 20) of patients, followed by the Sinopharm (20.0%, *n* = 7) and AstraZeneca (20.0%, *n* = 7) vaccines. The injection site was evenly distributed between the left and right shoulders, with 8.6% (*n* = 3) receiving injections in both shoulders. Approximately half of the patients (51.4%, *n* = 18) did not receive any treatment. Analgesic use was reported by 40% (*n* = 14) and physiotherapy was undertaken by 8.6% (*n* = 3). At the time of data collection, 22.9% (*n* = 8) of the patients still experienced SIRVA. Additionally, a significant majority (82.9%, *n* = 29) expressed hesitation about receiving future vaccinations.

### 3.2. Symptomatology of the First Vaccine Dose

Twenty-three patients developed symptoms after both doses, with an additional four experiencing symptoms after the first dose only and another eight after the second dose only. Following the first dose of the COVID-19 vaccine (Table 2), the most commonly reported symptom was pain (*n* = 14, 40%), followed by fever and numbness, each reported by 14.3% (*n* = 5) of patients. Other symptoms included flu-like symptoms (8.6%, *n* = 3), headache (8.6%, *n* = 3), limited range of motion (5.7%, *n* = 2), and malaise or general weakness (5.7%, *n* = 2).

The onset of symptoms occurred immediately after vaccination in 28% (*n* = 7) of symptomatic patients, within 24 h in 60% (*n* = 15), and within one week in 12% (*n* = 3). Regarding the duration of the symptoms, 52% of patients (*n* = 13) had symptoms lasting 1 to 7 days and only two patients experienced symptoms for more than a month. Treatment following the first dose was minimal. Most patients (62.9%, *n* = 22) did not receive any treatment. Analgesics were used by 34.3% (*n* = 12) of patients and physiotherapy was undertaken by one patient.

### 3.3. Symptomatology of the Second Vaccine Dose

After receiving the second vaccine dose (Table 2), pain remained the most prevalent symptom, reported by 51.4% (*n* = 18) of patients. Numbness was experienced by 14.3% (*n* = 5), fever by 11.4% (*n* = 4), both headache and general weakness by 8.6% (*n* = 3) of patients, and limited range of motion by 5.7% (*n* = 2).

The onset of symptoms post-second-dose occurred immediately in 16.7% (*n* = 4) of patients, within 24 h in 58.3% (*n* = 14), within one week in 12.5% (*n* = 3), and after more than one week in another 12.5% (*n* = 3). Symptom duration varied, with 41.7% (*n* = 10) experiencing them for 1 to 7 days; 4.2% (*n* = 1) for 1 to 4 weeks; and, notably, 25% (*n* = 6) reporting symptoms lasting more than a month. Over half of the patients (57.1%, *n* = 20) did not receive any treatment. Analgesics were used by 37.1% (*n* = 13) of patients and physiotherapy was undertaken by two patients.

### 3.4. MRI Findings

MRI evaluations were conducted on thirty-eight shoulders from thirty-five patients, including three patients who experienced SIRVA in both shoulders. The median interval between vaccination and MRI scans was 6 months (IQR = 3−12). Table 3 shows the differences in MRI findings between those who still have the symptoms and those who do not.

**Figure 1:**
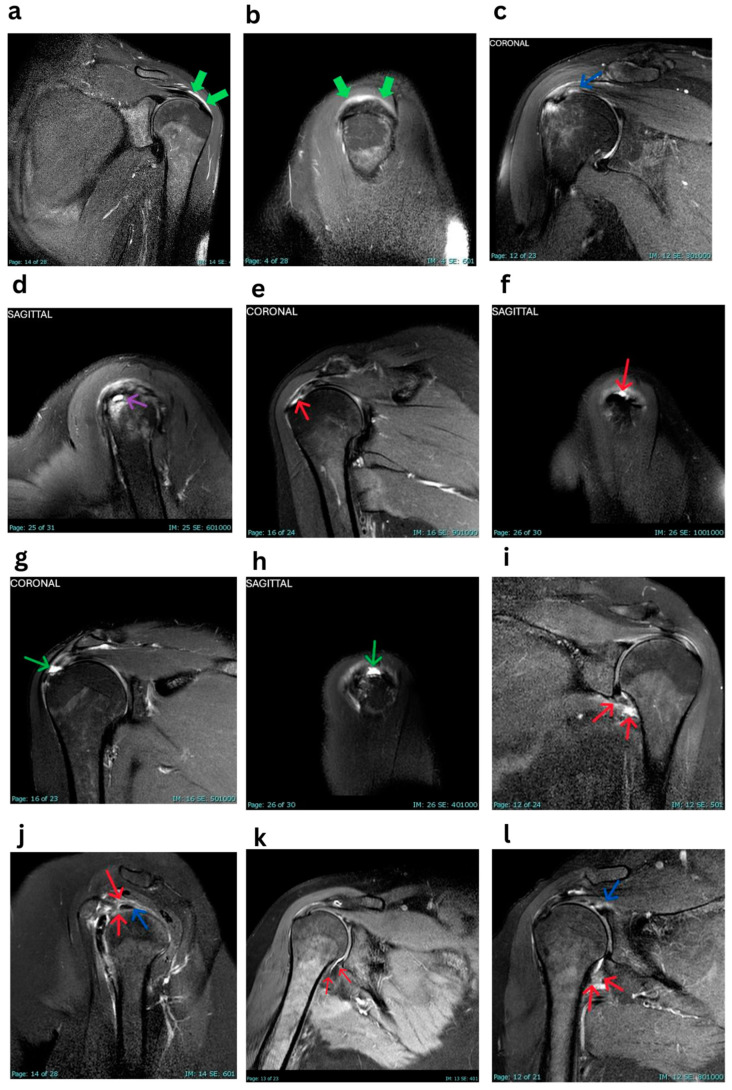
MRI findings. Thickened edematous subacromial-subdeltoid bursa in (**a**) coronal and (**b**) sagittal T2-SPAIR MR images (thick green arrows). Rotator cuff disease in (**c**) coronal and (**d**) sagittal (supraspinatus tendinopathy, blue arrow in (**c**), and purple arrow in (**d**)), and partial thickness tear in (**e**) coronal and (**f**) sagittal (red arrows). Full thickness tear in (**g**) coronal and (**h**) sagittal (thin green arrows). Thickened edematous glenohumeral joint capsule at (**i**) the axillary pouch (red arrows) and (**j**) rotator cuff interval (red arrows), indicating adhesive capsulitis. Note the intra-articular portion of the long head of the biceps tendon in (**j**) (blue arrow) and how it is clearly visible as a signal void structure in a background of the edematous capsule of the high T2 signal. Interval development of adhesive capsulitis after two years and eight months at the (**k**) initial and (**l**) follow-up MRI studies. In addition to edema at the axillary pouch in the follow-up images (red arrows in (**l**)), note the edema adjacent to the superior labrum (blue arrow in (**l**)) where the capsule also attaches.

The most prevalent MRI finding was subacromial bursitis (Figure 1a,b), observed in 89.5% (*n* = 34) of the shoulders examined. Erosions were detected in 63.2% (*n* = 24) of cases. Tendonitis was identified in 47.4% (*n* = 18) of shoulders (Figure 1c,d), predominantly affecting the supraspinatus tendon (39.5%, *n* = 15). The infraspinatus tendon was involved in 5.3% (*n* = 2) of cases and the long head of the biceps tendon in 7.9% (*n* = 3). Rotator cuff tears were present in 36.8% (*n* = 14) of shoulders (Figure 1e–h), with partial-thickness tears in 23.7% (*n* = 9) and full-thickness tears in 13.2% (*n* = 5), often alongside acute inflammatory signs. Changes in adhesive capsulitis were observed in 36.8% (*n* = 14) of cases (Figure 1i,j). Muscle edema was identified in 10.5% (*n* = 4) of patients. Joint effusion was detected in 23.7% (*n* = 9) of shoulders. None of the patients showed signs of avascular necrosis, axillary lymphadenopathy, or cartilage defects.

### 3.5. MRI Findings and Associations

The analysis of associations between MRI findings and patient characteristics revealed several significant relationships (Table 4). Notably, the presence of tendonitis was significantly associated with older age. Patients diagnosed with tendonitis had a higher mean age of 57.1 ± 7.4 years compared to 50.9 ± 8.8 years in those without tendonitis (*p* = 0.024). Additionally, tendonitis was significantly linked to the type of vaccine received (*p* = 0.033). A higher proportion of patients with tendonitis had received the AstraZeneca vaccine (38.9%) compared to those without tendonitis (5.3%), suggesting a potential association between this vaccine type and tendon inflammation.

Subacromial bursitis demonstrated significant associations with both the female gender and higher BMI. Females were more likely to exhibit subacromial bursitis than males (64.7% vs. 35.3%; *p* = 0.013). Moreover, patients with subacromial bursitis had a higher mean BMI than those without bursitis (29.1 ± 4.5 kg/m^2^ vs. 23.6 ± 2.1 kg/m^2^; *p* = 0.023, respectively).

The occurrence of adhesive capsulitis was significantly associated with the type of vaccine administered (*p* = 0.029). Patients with adhesive capsulitis were more likely to have received the Sinopharm vaccine (42.9%) compared to those without adhesive capsulitis (8.7%). Additionally, patients with adhesive capsulitis (AC) reported headaches more frequently (35.7%) than those without (8.3%), indicating a significant association between adhesive capsulitis and headache occurrence (*p* = 0.036).

### 3.6. Persistence of Symptoms

Table A1 shows the differences in patient characteristics between those who still have the symptoms and those who do not. Patients with persistent symptoms were significantly younger (<55 years: 87.5% vs. 44.4%, *p* = 0.032) and more likely to be female (87.5% vs. 44.4%, *p* = 0.032) compared to those without symptoms. Non-diabetic status was associated with ongoing symptoms (100% vs. 55.6%, *p* = 0.020). Right-sided injections were more common among patients with persistent symptoms (87.5% vs. 33.3%, *p* = 0.025). All patients with persistent symptoms reported pain (100% vs. 48.2%, *p* = 0.009). Additionally, experiencing symptoms after the second vaccine dose was linked to persistent symptoms (100% vs. 59.3%, *p* = 0.029).

There were no significant differences in MRI findings between patients who continued to experience symptoms and those whose symptoms had resolved. However, there was a trend towards a higher incidence of tendonitis among patients with persistent symptoms (75% vs. 40%, *p* = 0.078). However, a comparative analysis was conducted for three patients who underwent initial and follow-up MRI scans to assess changes in shoulder pathology over time after COVID-19 vaccination (Table A2).

## 4. Discussion

In this retrospective cohort of 35 adults who developed new shoulder symptoms within six weeks of COVID-19 vaccination, subacromial-subdeltoid (SASD) bursitis was the dominant MRI abnormality, present in most examined shoulders. Tendinopathy was identified in roughly half of the cases, chiefly involving the supraspinatus, whereas rotator cuff tears and adhesive capsulitis each appeared in about one-third. Significant associations were observed between tendinopathy and both older age and receipt of the AstraZeneca vaccine; SASD bursitis and female sex combined with being overweight; and adhesive capsulitis and receipt of the Sinopharm vaccine. Persistent symptoms at follow-up were more common in younger women and after right-sided injections, with a non-significant trend toward co-existing tendinopathy.

SIRVA was initially mentioned in the literature in 2010 by the Vaccine Injury Compensation Program in the United States, though it was a medicolegal term, reflecting the rising number of claims back then for shoulder complaints following immunization [8]. It was not until the COVID-19 pandemic that SIRVA emerged strongly as a true diagnosis following the mass vaccination campaigns worldwide. However, despite the dramatic surge in publications related to SIRVA ever since, there has been a lack of large-scale studies about the imaging features of this disease and their clinic–demographic associations being mainly dominated by case reports or case series.

Donners et al. had the largest case series describing MRI findings of SIRVA [9]. They evaluated contrast-enhanced shoulder MRI images for nine patients diagnosed with chronic SIRVA for several predefined sets of potential findings. To the best of our knowledge, their study is the only one aiming primarily to document the imaging features of this disease. However, unlike ours, their article did not correlate these findings with patients’ clinical and/or demographic characteristics. Furthermore, we successfully evaluated imaging findings in 38 shoulders of 35 patients collected during the initial year following the start of the COVID-19 vaccination campaign at our center between May 2021 and May 2022, making our patient cohort more representative of the acute/subacute stage of the disease.

Cook described the first case report with SIRVA back in 2014 [10]. His patient was diagnosed with SASD bursitis following influenza vaccine administration. He anticipated that this could have been prevented if an evidence-based protocol had been conducted to avoid potential injury to some important regional anatomical structures, with the SASD bursa on the top of the list [11] if the vaccine administrator aimed “too high” [4]. Inflammation of the SASD bursa in patients with SIRVA has been consistently demonstrated in the literature, resonating with our results that showed that 89.5% of patients developed this pathology as the most common imaging finding in our cohort (Figure 1a,b). However, this does not coincide with the results of Donners et al., who only found one patient with SASD bursitis [9], probably due to their small sample size. Our study also found that SASD bursitis is more common in females and patients with a higher BMI. A thicker subcutaneous tissue could explain the gender and weight difference in patients with SIRVA-related bursitis.

The second-most-common MRI finding in our study was rotator cuff disease, represented by tendinopathy and partial- or full-thickness tears (Figure 1c–f), with an accumulated occurrence of about 84.2% in our patient cohort. Bass et al. reported tendinopathy in 19 patients (20%) in their systematic review [12], which is notably lower than our findings when excluding cuff tears (47.4%). Tendinopathy commonly occurs with advancing age. The mean age in both studies was almost identical (53.5 and 53.6 years). In our study, the association between age and tendinopathy was statistically significant. Additionally, we noticed a correlation between tendinopathy and the AstraZeneca vaccine, although the underlying cause of this association remains unclear. It is difficult to determine whether these findings were merely caused by the vaccine or simply reflective of age-related changes. This is further supported by the high prevalence of greater/lesser tuberosity erosion in our cohort (63.2%), which usually corresponds to chronic cuff disease (Figure 1c,d).

Regarding cuff tears, the supraspinatus tendon was the most commonly affected (39.5%). However, any tendon can be affected depending on the needle trajectory and cuff positioning in space at the time of immunization. Natanzi et al. reported two cases where the teres minor tendon was affected; only such cases were documented in the literature [13]. This rarity may suggest that the teres minor tendon may be less commonly implicated and potentially safe during vaccine administration. In our study, fifteen tears were documented in shoulders that simultaneously exhibited acute inflammatory signs (subacromial bursitis, peri-tendinous oedema, or high-signal tendon fraying) and none of the affected patients reported interval trauma or unusual mechanical loading. We, therefore, interpret these ruptures as pre-existing degenerative foci that became clinically manifest in the inflammatory milieu of SIRVA, rather than new lesions caused directly by the injection.

Although a retrospective design cannot prove cause-and-effect, several design features and findings strengthen the inference that vaccination precipitated the observed pathology. First, we excluded every patient with documented shoulder disease or recent trauma. Second, inclusion required symptom onset within six weeks of vaccination, sharply limiting alternative timelines. Third, the MRI abnormalities consistently co-existed with acute inflammatory markers, subacromial fluid, marrow oedema, and high-signal tendon fraying, findings rarely present in silent, chronic degeneration. Together, these measures make an incidental origin unlikely, yet they do not constitute definitive proof of causality.

One of the most devastating shoulder pathologies that can happen following vaccination is AC, or what is globally known as the “Frozen Shoulder”. This disease is characterized by excessive scarring of the glenohumeral capsule, leading to painful limitation in range of motion [14]. On MRI, it usually presents as increased thickness and a T2 signal in the coracohumeral ligament and axillary capsule (Figure 1g,h), sometimes with obliteration of the subcoracoid fat triangle [15]. In the systematic review conducted by Bass et al. [12], AC was the most common pathology found in patients with COVID-19-associated SIRVA, constituting 37.9% of imaging-proven AC. This is almost identical to our results (36.8%), which also showed a significant association between AC and the Sinopharm vaccine and headache complaints. The latter association can be explained by the prolonged duration of the disease, resulting in stiffness in muscles connecting the shoulder girdle and calvarium, such as the trapezius, though this has not been explored further in our study. If left without management, SIRVA-related AC can lead to prolonged shoulder pain, loss of mobility, and chronic dysfunction, which may persist for years [1], impacting quality of life [12]. Two out of three patients who had follow-up MRI in our cohort (66%) had interval development of AC (Figure 1k,l). Moreover, two out of eight patients with persistent symptoms after 2–3 years of vaccination (25%) had AC. These two last numbers highlight the debilitating nature of AC in SIRVA cases and the importance of its early recognition and initiation of therapy to improve long-term prognosis.

Other potential structures that can be injured during vaccination include, but are not limited to, the anterior branches of the axillary [16] and radial [17] nerves, the humeral head [18], and the glenohumeral joint. Nerve injury usually presents clinically as numbness or paresthesia. However, it barely has imaging findings, especially if the field of view is limited to a particular area, such as the shoulder, as in our study. Humeral head injury may result in avascular necrosis or cartilage defects. Neither of these findings were seen in our patient cohort, despite being previously mentioned in the literature [9], reflecting the rarity of their occurrence. Finally, axillary lymphadenopathy and calcific tendinitis are other shoulder entities in SIRVA but none of our patients had these [9,19].

Limitations of this study include the retrospective single-center design, the absence of baseline MRI, and the use of a non-enhanced shoulder MRI protocol. The retrospective approach, however, enabled the timely identification of real-world cases needed to meet the study objective while stringent exclusion criteria and a narrow post-vaccination window mitigated potential bias. Additionally, symptom-based case finding, dictated by the clinical case definition of SIRVA, may have missed silent imaging-positive cases, although concordant MRI pathology substantially reduced diagnostic uncertainty. Furthermore, because the radiologists knew the patients presented with post-vaccination shoulder complaints, the potential for confirmation bias cannot be fully excluded. Future studies may benefit from prospective, blinded reading protocols and, where clinically indicated, contrast-enhanced imaging to capture more nuanced pathological changes. The analysis of associations between MRI findings and patient characteristics revealed several nominally significant relationships. However, because multiple comparisons were performed, the *p*-values reported are unadjusted for potential inflation of the Type I error. Consequently, these findings should be interpreted with caution and they serve primarily as an exploratory assessment. Additionally, the absence of a formal control group represents a key limitation in interpreting our MRI findings. Although patients with previously documented shoulder pathology were excluded, asymptomatic degenerative changes, particularly among older adults, can manifest as erosions, tendonitis, or partial rotator cuff tears and may not necessarily reflect vaccine-related injury. As a result, it is difficult to determine whether the observed MRI findings truly exceed the background prevalence of shoulder abnormalities in a matched, non-vaccinated or asymptomatic vaccinated population. To confirm our results, future studies with larger samples and a case-control design, with individuals of comparable age and demographic profiles, could help establish a more definitive link between COVID-19 vaccine administration and shoulder pathology while accounting for common degenerative changes.

## 5. Conclusions

Subacromial-subdeltoid bursitis was the predominant MRI finding in post-vaccination SIRVA. Tendinopathy, chiefly of the supraspinatus, may be associated with older age and receipt of the AstraZeneca vaccine, whereas bursitis correlated with female sex and higher BMI. Adhesive capsulitis occurred in roughly one-third of shoulders and may be linked to the Sinopharm vaccine. These imaging–demographic patterns delineate the spectrum of COVID-19-associated SIRVA and may assist clinicians in earlier recognition and tailored management of affected patients.

## Figures and Tables

**Table 1:** Patient demographics and vaccination characteristics.

	Total *n* = 35
Age, mean (SD)	53.6 (9.0)
Age groups	
<55 years	19 (54.3)
≥55 Years	16 (45.7)
Gender	
Male	16 (45.7)
Female	19 (54.3)
Weight, mean (SD)	78.5 (13.0)
Height, mean (SD)	166.3 (9.5)
BMI, mean (SD)	28.4 (4.8)
BMI categories	
Normal (18.5–24.9)	7 (20)
Overweight (25–29.9)	15 (42.9)
Obese (≥30)	13 (37.1)
Regular physical activity	
No	19 (54.3)
Sometimes	10 (28.6)
Yes	6 (17.1)
Diabetes	
No	23 (65.7)
Yes	12 (34.3)
Type of vaccine	
Pfizer	20 (57.1)
Sinopharm	7 (20)
AstraZeneca	7 (20)
Others	1 (2.9)
Side injected	
Left	16 (45.7)
Right	16 (45.7)
Both	3 (8.6)
Treatment received	
None	18 (51.4)
Analgesics	14 (40)
Physiotherapy	3 (8.6)
Still have symptoms	
Yes	8 (22.9)
No	27 (77.1)
Hesitant to take vaccines in the future	
Yes	29 (82.9)
Maybe	3 (8.6)
No	3 (8.6)

**Table 2:** Symptomatology of the first and second vaccine doses.

	First Vaccine Dose Freq. (%)	Second Vaccine Dose Freq. (%)
Side injected		
Left	18 (51.4)	20 (57.1)
Right	17 (48.6)	15 (42.9)
Symptom		
Pain	14 (40)	18 (51.4)
Fever	5 (14.3)	4 (11.4)
Numbness	5 (14.7)	5 (14.3)
Flu-like	3 (8.8)	1 (2.9)
Headache	3 (8.6)	3 (8.6)
Limited ROM	2 (5.7)	2 (5.7)
Malaise/G Weakness	2 (5.7)	3 (8.6)
Slow limb motion	1 (2.9)	0
Sweating	1 (2.9)	0
SOB	1 (2.9)	0
Dizziness	1 (2.9)	0
Heaviness	0	1 (2.9)
Start of symptoms		
Immediately	7 (28.0)	4 (16.7)
Within 24 h	15 (60.0)	14 (58.3)
Within 1 week	3 (12.0)	3 (12.5)
Unknown	10 (28.6)	3 (12.5)
Duration of symptoms		
less than 24 h	8 (32.0)	7 (29.2)
1–7 days	13 (52.0)	10 (41.7)
1–4 weeks	2 (8.0)	1 (4.2)
More than a month	2 (8.0)	6 (25.0)
Unknown	10 (28.6)	11 (31.4)
Treatments		
None	22 (62.9)	20 (57.1)
Analgesics	12 (34.3)	13 (37.1)
Physiotherapy	1 (2.9)	2 (5.7)

**Table 3:** Differences in MRI findings between those who still have the symptoms and those who do not.

MRI Findings		Still Have Symptoms		
	Total	No	Yes	*p*-Value
Erosions	24 (63.2)	19 (63.3)	5 (62.5)	0.965
Bone marrow edema	3 (7.9)	3 (10)	0 (0)	0.351
Tendonitis	18 (47.4)	12 (40)	6 (75)	0.078
Supraspinatus	15 (39.5)	10 (33.3)	5 (62.5)	0.134
Infraspinatus	2 (5.3)	2 (6.7)	0 (0)	0.453
LHBT	3 (7.9)	2 (6.7)	1 (12.5)	0.587
Muscle edema	4 (10.5)	4 (13.3)	0 (0)	0.275
Adhesive capsulitis	14 (36.8)	12 (40)	2 (25)	0.435
Subacromial bursitis	34 (89.5)	27 (90)	7 (87.5)	0.838
Rotator cuff tear	14 (36.8)			
Partial thickness	9 (23.7)	7 (23.3)	2 (25)	0.922
Full thickness	5 (13.2)	5 (16.7)	0 (0)	0.215
Effusion	9 (23.7)	7 (23.3)	2 (25)	0.922

LHBT, long head of the biceps tendon.

**Table 4:** Association between study variables and MRI findings.

	Erosions			Tendonitis			SAB Bursitis			RC Tears			**Adhesive Capsulitis**		
	No	Yes	*p*-Value	No	Yes	*p*-Value	No	Yes	*p*-Value	No	Yes	*p*-Value	**No**	**Yes**	***p*-Value**
Age mean (SD)	52 (10)	55 (8)	0.21	51 (9)	57 (7)	0.024	53 (22)	54 (6)	0.8	53 (10)	56 (6)	0.3	52 (9)	57 (7)	0.15
Age groups			0.8			0.8			0.24			0.4			0.8
<55 years	7 (50)	13 (54)		11 (55)	9 (50)		1 (25)	19 (56)		14 (58)	6 (43)		13 (54)	7 (50)	
≥55 Years	7 (50)	11 (46)		9 (45)	9 (50)		3 (75)	15 (44)		10 (42)	8 (57)		11 (46)	7 (50)	
Gender			0.5			0.3			0.013			0.2			0.5
Female	7 (50)	15 (63)		10 (50)	12 (67)		0 (0)	22 (65)		12 (50)	10 (71)		13 (54)	9 (64)	
Male	7 (50)	9 (38)		10 (50)	6 (33)		4 (100)	12 (35)		12 (50)	4 (29)		11 (46)	5 (36)	
BMI mean (SD)	29 (6)	29 (4)	0.9	28 (4)	30 (5)	0.15	24 (2)	29 (5)	0.023	29 (5)	28 (3)	0.9	29 (5)	28 (3)	0.4
BMI categories			0.13			0.5			0.007			0.2			0.9
Normal (18.5–24.9)	4 (29)	3 (13)		5 (25)	2 (11)		3 (75)	4 (12)		6 (25)	1 (7)		4 (17)	3 (21)	
Overweight (25–29.9)	3 (21)	13 (54)		7 (35)	9 (50)		1 (25)	15 (44)		8 (33)	8 (57)		10 (42)	6 (43)	
Obese (≥30)	7 (50)	8 (33)		8 (40)	7 (39)		0 (0)	15 (44)		10 (42)	5 (36)		10 (42)	5 (36)	
Regular physical activity			0.9			0.3			0.8			0.6			0.4
No	8 (57)	12 (50)		12 (60)	8 (44)		2 (50)	18 (53)		14 (58)	6 (43)		13 (54)	7 (50)	
Sometimes	4 (29)	8 (33)		4 (20)	8 (44)		1 (25)	11 (33)		7 (29)	5 (36)		6 (25)	6 (43)	
Yes	2 (14)	4 (17)		4 (20)	2 (11)		1 (25)	5 (15)		3 (13)	3 (21)		5 (21)	1 (7)	
Diabetes			0.6			0.4			0.5			0.4			0.6
No	10 (71)	15 (63)		12 (60)	13 (72)		2 (50)	23 (68)		17 (71)	8 (57)		15 (63)	10 (71)	
Yes	4 (29)	9 (38)		8 (40)	5 (28)		2 (50)	11 (33)		7 (29)	6 (43)		9 (38)	4 (29)	
Type of vaccine			0.6			0.033			0.3			0.7			0.029
Pfizer	7 (54)	14 (58)		14 (74)	7 (39)		2 (50)	19 (58)		14 (61)	7 (50)		14 (61)	7 (50)	
Sinopharm	2 (15)	6 (25)		4 (21)	4 (22)		2 (50)	6 (18)		5 (22)	3 (21)		2 (9)	6 (43)	
AstraZeneca	4 (31)	4 (17)		1 (5)	7 (39)		0 (0)	8 (24)		4 (17)	4 (27)		7 (30)	1 (7)	
Side injected			0.5			1.0			0.3			0.2			1.0
Left	6 (43)	13 (54)		10 (50)	9 (50)		1 (25)	18 (53)		14 (58)	5 (36)		12 (50)	7 (50)	
Right	8 (57)	11 (46)		10 (50)	9 (50)		3 (75)	16 (47)		10 (42)	9 (64)		12 (50)	7 (50)	
Symptoms															
Pain	8 (57)	14 (58)	0.9	13 (65)	9 (50)	0.3	2 (50)	20 (59)	0.7	14 (58)	8 (57)	0.9	15 (63)	7 (50)	0.5
Fever	3 (21)	4 (17)	0.7	4 (20)	3 (17)	0.8	1 (25)	6 (18)	0.7	6 (25)	1 (7)	0.17	4 (17)	3 (21)	0.7
Numbness	1 (7)	5 (21)	0.3	4 (20)	2 (11)	0.5	0 (0)	6 (18)	0.4	2 (8)	4 (29)	0.1	3 (13)	3 (21)	0.5
Headache	3 (21)	4 (17)	0.7	4 (20)	3 (17)	0.8	0 (0)	7 (21)	0.3	4 (17)	3 (21)	0.7	2 (8)	5 (36)	0.036
Limited ROM	1 (7)	2 (8)	0.9	1 (5)	2 (11)	0.5	0 (0)	3 (9)	0.5	2 (8.3)	1 (7)	0.9	1 (4)	2 (14)	0.3
Still have symptoms			0.9			0.08			0.8			0.4			0.4
Yes	3 (21)	5 (21)		2 (10)	6 (33)		1 (25)	7 (21)		6 (25)	2 (14)		6 (25)	2 (14)	
No	11 (79)	19 (79)		18 (90)	12 (67)		3 (75)	27 (79)		18 (75)	12 (86)		18 (75)	12 (86)	
Hesitant to take the vaccine			0.9			0.7			0.004			0.13			0.9
Yes	12 (86)	20 (83)		17 (85)	15 (83)		2 (50)	30 (88)		18 (75)	14 (100)		2 (8)	1 (7)	
Maybe	1 (7)	2 (8)		2 (10)	1 (6)		0 (0)	3 (9)		3 (13)	0 (0)		2 (8)	1 (7)	
No	1 (7)	2 (8)		1 (5)	2 (11)		2 (50)	1 (3)		3 (13)	0 (0)		2 (8)	1 (7)	
Treatments			0.9			0.6			0.6			0.30			0.5
None	7 (50)	13 (54)		9 (45)	11 (61)		3 (75)	17 (50)		11 (49)	9 (64)		13 (54)	7 (50)	
Analgesics	6 (43)	9 (38)		9 (45)	6 (33)		1 (25)	14 (41)		10 (42)	5 (36)		10 (42)	5 (36)	
Physiotherapy	1 (7)	2 (8)		2 (10)	1 (6)		0 (0)	3 (9)		3 (13)	0 (0)		1 (4)	2 (14)	

All *p*-values presented are unadjusted for multiple comparisons. Given the exploratory nature of this study, no formal correction was applied.

## Data Availability

The data supporting the findings of this study are not openly available but are available from the corresponding author upon reasonable request.

## Abbreviations

The following abbreviations are used in this manuscript:

SIRVA	Shoulder Injury Related to Vaccine Administration
MRI	Magnetic resonance imaging
BMI	Body mass index
SASD	Subacromial/subdeltoid
AC	Adhesive capsulitis

## Appendix A

**Table A1:** Differences in the patient characteristics and MRI findings between those who still have the symptoms and those who do not.

	Still Have Symptoms		
	No	Yes	*p*-Value
Age mean (SD)	55.0 (8.0)	49.1 (11.0)	0.106
Age groups			0.032
<55 years	12 (44.4)	7 (87.5)	
≥55 Years	15 (55.6)	1 (12.5)	
Gender			0.032
Female	12 (44.4)	7 (87.5)	
Male	15 (55.6)	1 (12.5)	
BMI mean (SD)	28.3 (4.4)	29.0 (6.3)	0.720
BMI categories			0.441
Normal (18.5–24.9)	6 (22.2)	1 (12.5)	
Overweight (25–29.9)	10 (37)	5 (62.5)	
Obese (≥30)	11 (40.7)	2 (25)	
Regular physical activity			0.109
No	17 (63)	2 (25)	
Sometimes	7 (25.9)	3 (37.5)	
Yes	3 (11.1)	3 (37.5)	
Diabetes			0.020
No	15 (55.6)	8 (100)	
Yes	12 (44.4)	0 (0)	
Type of vaccine			0.257
Pfizer	14 (53.9)	6 (75)	
Sinopharm	7 (26.9)	0 (0)	
AstraZeneca	5 (19.2)	2 (25)	
Side injected			0.025
Left	15 (55.6)	1 (12.5)	
Right	9 (33.3)	7 (87.5)	
Both	3 (11.1)	0 (0)	
With any symptom 1st			0.252
Yes	18 (66.7)	7 (87.5)	
No	9 (33.3)	1 (12.5)	
With any symptom 2nd			0.029
Yes	16 (59.3)	8 (100)	
No	11 (40.7)	0 (0)	
With any symptom any dose			0.058
Yes	18 (66.7)	8 (100)	
No	9 (33.3)	0 (0)	
Symptoms			
Pain	13 (48.2)	8 (100)	0.009
Fever	5 (18.5)	1 (12.5)	0.692
Numbness	5 (18.5)	1 (12.5)	0.692
Headache	4 (14.8)	1 (12.5)	0.869
Limited ROM	1 (3.7)	2 (25)	0.059
Hesitant to take vaccine			0.576
Yes	22 (81.5)	7 (87.5)	
Maybe	3 (11.1)	0 (0)	
No	2 (7.4)	1 (12.5)	
Treatments			0.234
None	16 (59.3)	2 (25)	
Analgesics	9 (33.3)	5 (62.5)	
Physiotherapy	2 (7.4)	1 (12.5)	

## Appendix B

**Table A2:** Comparison of MRI findings between initial and follow-up imaging.

Patient	Initial MRI Date	Follow-Up MRI Date	Initial MRI Findings	Follow-Up MRI Findings	Improvements	Deteriorations
Patient 1	5 January 2022	9 July 2023	- Erosions in the greater tuberosity - Tendonitis of the supraspinatus, infraspinatus, and long head of biceps tendon (LHBT) - Muscle edema present - Subacromial bursitis presentNo rotator cuff tear	- Erosions persisted - Continued tendonitis of the supraspinatus, infraspinatus, and LHBT - Muscle edema resolved - Subacromial bursitis resolved - New axillary capsule involvement - New full-thickness tear of the supraspinatus tendon - No effusion	- Resolution of muscle edema - Resolution of subacromial bursitis	- Development of axillary capsule involvement - Onset of full-thickness rotator cuff tear
Patient 2	25 August 2021	21 April 2024	- Erosions in the lesser tuberosity - Rotator cuff interval involvement - Subacromial bursitis present - Partial-thickness tear of the supraspinatus tendon - No muscle edema	- Erosions persisted - Rotator cuff interval involvement resolved - Subacromial bursitis resolved - Rotator cuff tear healed - New muscle edema developed	- Resolution of rotator cuff interval involvement - Resolution of subacromial bursitis - Healing of rotator cuff tear	- Development of muscle edema
Patient 3	15 February 2021	30 October 2023	- Tendonitis of the supraspinatus tendon - Subacromial bursitis present - No rotator cuff interval or axillary capsule involvement - No effusion	- Tendonitis resolved - New involvement of the rotator cuff interval and axillary capsule - Effusion - Development of calcific tendonitis - Subacromial bursitis resolved	- Resolution of supraspinatus tendonitis - Resolution of subacromial bursitis	- Development of rotator cuff interval and axillary capsule involvement - Onset of effusion and calcific tendonitis
